# An Update Review on the Paneth Cell as Key to Ileal Crohn's Disease

**DOI:** 10.3389/fimmu.2020.00646

**Published:** 2020-04-15

**Authors:** Jan Wehkamp, Eduard F. Stange

**Affiliations:** University of Tübingen, Medizinische Klinik I, Tübingen, Germany

**Keywords:** Paneth cell, Crohn's disease, ileum, bacterial recognition, autophagy, endosomal stress, necroptosis

## Abstract

The Paneth cells reside in the small intestine at the bottom of the crypts of Lieberkühn, intermingled with stem cells, and provide a niche for their neighbors by secreting growth and Wnt-factors as well as different antimicrobial peptides including defensins, lysozyme and others. The most abundant are the human Paneth cell α-defensin 5 and 6 that keep the crypt sterile and control the local microbiome. In ileal Crohn's disease various mechanisms including established genetic risk factors contribute to defects in the production and ordered secretion of these peptides. In addition, life-style risk factors for Crohn's disease like tobacco smoking also impact on Paneth cell function. Taken together, current evidence suggest that defective Paneth cells may play the key role in initiating inflammation in ileal, and maybe ileocecal, Crohn's disease by allowing bacterial attachment and invasion.

## Introduction

Crohn's disease was originally described and finally established (1) as a chronic ileal inflammation leading to strictures and finally resection of the involved segment. Over time it became evident that there was also a form of colonic Crohn's disease (2) and actually the disease may involve all parts of the gastrointestinal tract from mouth to anus. The respective localization is remarkably stable in a given patient whereas the disease behavior may advance from a mere inflammatory process to strictures as well as fistulas penetrating the gut wall (3). This categorization into ileal, colonic, and combined, usually ileocecal or ileocolonic localization also has a genetic background (4). Originally thought to be an autoimmune type of disease, the current view is that the immune response is directed against and induced by the intestinal microbiome and the gut inflammation is at least in part a collateral damage of this interaction (5). The separate localization types imply that if indeed Crohn's disease was characterized by a defective barrier toward intestinal microbes (6), the cellular and molecular basis of this defect was likely to be local and differ between ileal and colonic Crohn's disease.

One possible explanation may be provided by the Paneth cell which resides predominantly in the small intestine, although it may also be induced by inflammation as a metaplastic cell in other parts of the intestine, such as in the colon. The history of this cell (7) dates back to 1872 when it was first observed by Schwalbe in Freiburg but described in more detail in 1888 by Josef Paneth in Vienna (who actually quoted Schwalbe and showed one of his pictures). It took nearly a century to elucidate the function of Paneth cells: in an exhaustive study on the Paneth cell in gastrointestinal disease published in 1969 it was still speculated that the granules contained a kind of zymogen, possibly a peptidase and was therefore involved in digestion (8). Finally, lysozyme was detected in Paneth cells of the small intestine (9), compatible with their now established role in bacterial killing. However, quantitatively and biologically the most important Paneth cell products are the antibacterial α-defensins, i.e., human defensin 5 (HD5) (10) and, to a lesser degree, human defensin 6 (HD6) (11). Apparently, the antibiotic peptides secreted form a chemical barrier preventing bacterial invasion and any defect in Paneth cell function may therefore compromise mucosal integrity. We therefore provocatively (and tongue in cheek) renamed this entity of ileal Crohn's disease as “Paneth's disease” (7) and ten years after it seems appropriate to look at the current state of the Paneth cell in Crohn's disease. Paneth cells are Janus-faced: they were given the title “maestros of the crypt” (12) but they may also be the culprits in Crohn's disease, hiding backstage behind the *T*-cells.

## Paneth Cell And Defensin Physiology

Located at the base of the crypts of Lieberkühn, the Paneth cells appear to serve a dual function: support of the surrounding LGR5 positive stem cells and antibacterial secretion. The first and quite essential role is based on the local secretion of trophic factors supporting the stem cell niche of neighboring crypt base columnar cells, from which all other small intestinal cell lineages originate (13). The trophic factors include epidermal growth factor, transforming growth factor α and Wnt3. Coculture of Paneth cells with stem cells is 10 times more efficient in the formation of organoids than single type stem cell cultures. This suggests an important role of this interaction also *in vivo*.

The limited population of about 5–15 Paneth cells per crypt is under strict control by a complex net of differentiation factors, the most important being the Wnt-factor TCF4 (also known as TCF7L2) (14). TCF4 drives both a stem cell/progenitor gene program and a Paneth cell maturation program. Indian hedgehog is another important mediator, that is secreted by mature Paneth cells and forms a feedback loop down-regulating differentiation from Paneth cell precursors (15). Finally, during mouse Paneth cell development colony stimulating factor-1 is important (16) as well as other downstream mediators of Wnt (17).

Following differentiation, Paneth cell granule secretion into the crypt lumen is governed by cholinergic and bacterial factors (18), probably mediated by NOD2 (19) and TLR9 (20). TLR (toll like receptor) signals are transferred through MyD88, limiting microbial adherence and invasion through Paneth cell direct sensing and antibacterial secretions (21). Interestingly, as shown in organoids only the apical and not the basolateral surface of Paneth cells was responsive to lipopolysaccharide or live bacteria (22). However, even simple molecules like butyric acid or leucine may induce Paneth cell α-defensin secretion (23). Another factor involved, especially in maintaining the α-defensin precursor activating enzyme MMP7 in the starving mouse is mTOR (24). However, regulation may also be independent of microbiota such as lymphocyte derived interleukins which trigger Paneth cells to secrete antibacterial peptide, in this case angiogenin 4 (25). In addition, it was recently shown that also monocytes may induce Paneth cell defensins, probably via Wnt-factors (26). Others emphasized the key role of interferon signaling in Paneth cell function (27), thereby affecting microbial ecology (28). It is conspicuous that the Paneth cell also seems to be the main source of IL17 (29) as well as TNF, a major inflammatory cytokine and therapeutic target in the intestine (30).

Notably, Paneth cells produce a whole array of antibacterial peptides in addition to the α-defensins and angiogenin, including lysozyme as mentioned above but also lectins like RegIIIα in man or RegIIIγ in the mouse as well as type II secretory phospholipase A2 (12). Nevertheless, the key antibacterials are the two α-defensins (31), with different main modes of action. HD5 is a direct antibacterial and, if the human gene is “knocked into” a mouse, this will then change its commensal microbiome composition (32) and the mouse becomes resistant to Salmonella infection (33): thus the host defensins select its commensal microbiota but also protect against invaders. HD5 peptide in the intestine is unstable, however, and may be degraded by proteases into up to 8, 000 new antimicrobial peptide combinations which dramatically increase the host's ability to control pathogens and commensals (34). In contrast, HD6 is rather stable and predominantly acts by forming peptide nanonets inhibiting bacterial movement (35) rather than direct killing. Killing is only observed upon chemical reduction of the peptide (36), similar to HBD1 (37). It should be noted that these α-defensins are not only observed in the crypts and lumen of the small intestine but in the mouse are also transported intact from the small intestine to the colonic lumen, suggesting an impact also on the colonic microbiome (38). In this species α-defensins are called cryptdins also exhibiting strong bactericidal activity (39). However, their primary function likely is the prevention of bacterial migration through the ileocecal valve from the colon into and up the small intestine, resulting in about 1000-fold lower bacterial counts in the terminal ileum compared to the colon.

## Paneth Cell Function In Crohn'S Disease

In a first series of ileal Crohn's disease patients from Germany both ileal HD5 and HD6 were diminished compared to controls (40) whereas those with unaffected ileum and colonic disease exhibited a normal expression. In the colon enhanced expression of both α-defensins reflected Paneth cell metaplasia. In a second series of American and German patients combined low HD5-expression and protein in the affected Crohn's ileum was confirmed, and this finding was shown to be independent of the degree of tissue inflammation, whereas IL-8 was directly related to inflammation (41). Concomitantly antimicrobial activity of ileal mucosa was compromised and all other non-defensin antimicrobial peptides measured including lysozyme or phospholipase A2 were in the normal range. This suggested that the relative defensin deficiency was the key to defective antibacterial activity. However, other antibacterials like angiogenin (42) may also have important roles.

In further investigations this diminished Paneth cell defensin expression was linked to the Wnt system, in particular TCF4 (43), LRP6, and TCF 1 (44). As mentioned above, monocytes may activate Paneth cells, probably through Wnt factors but this mechanism was shown to be defective in monocytes from Crohn's disease (26). Thus, there is a direct link between bone marrow derived and Paneth cells controlling the microbiome.

In a study from Australia low HD5 expression was confirmed but not independent of inflammation (45). The authors explained their findings by a loss of surface epithelium during inflammation, i.e., in ulcerated areas inflammation may indeed also affect the Paneth cell area. Avoiding problems of varying biopsy sites an English study quantitated HD5 in ileal effluents and found these to be reduced in Crohn's patients. This occurred without apparent inflammation compared to controls, but levels were particularly low if there was active disease (46). Moreover, HD5 in Crohn's disease gut lumen persisted in a complex of trypsin and chymotrypsin as well as in an immature precursor form, probably compromising its antibacterial activity. It is conceivable that the multiple proteolytic imbalances described in Crohn's disease affect the intraluminal degradation of HD5 mentioned above (33). In uninvolved Crohn's jejunum HD6 expression was diminished but not HD5 (47). More recently it was demonstrated that the HD5-gene showed a higher methylation status in Crohn's disease, regardless of inflammation, although the number of HD5 positive Paneth cells was normal (48). Thus, this apparently permanent gene methylation may be important in silencing the HD5 gene.

In an initial pediatric cohort both HD5 and TCF4 were low and correlated (49), whereas in another study of children with Crohn's disease only ileal TCF4 was diminished but not HD5 (50). Interestingly, in a very recent large study looking at a global pattern of ileal gene expression low HD5 expression was observed specifically in older children of 10 years age and above while younger children did not exhibit this decrease (51). Therefore, the authors suggested that this defensin deficiency may explain the rapid rise of IBD during puberty. Finally, also in pediatric patients, and independent of the genetic associations with Paneth cell defects discussed below, a phenomenon related to autophagy induced crinophagy was described specifically in ileal Crohn's disease (52). This was independent of inflammation and resulted in a significant decrease in the number of secretory granules. Taken together, despite some inconsistencies and remaining controversy, the current evidence, independent of the genetic studies discussed below, relates compromised Paneth cell function and even morphology to ileal Crohn's disease both in (older) pediatric and adult populations. However, to prove a primary role of such a defect, the genetic basis has to be clarified.

In addition to the changes in α-defensins, interesting observations suggest that HBD-3 peptide expression (but not mRNA) is actually increased in ileal Crohn's disease and it is relocated from the luminal surface and Paneth cell granules to the basolateral surface and the lamina propria (53).

## The Paneth Cell And Genetic Links To Crohn'S Disease

NOD2 (nucleotide binding oligomerization domain 2) came into the focus since the revolutionary observation that single nucleotide polymorphisms in various genes are related to the risk of Crohn's disease, in particular ileal Crohn's disease (54, 55). This first and relevant link is an intracellular receptor for bacterial derived muramyldipeptide (MDP)and is expressed in several cell types including monocytes and, notably, the Paneth cell (19). After binding to MDP, NOD2 oligomerizes and binds to the serine-threonine kinase RIP2 and finally the complex mediates the signal to the IKK complex which then activates NFκB. Expression of NOD2 and the NOD2/RIP2 complex is enhanced in Crohn's disease (19, 56) and, somewhat paradoxically, NOD2 may actually suppress HD5 and HD6 formation in cultured Caco2 cells differentiated to Paneth like cells through action of FGF9 (57). On the other hand, MDP-NOD2 stimulation induced the defensin HNP-1 (human neutrophil peptide 1) in Caco-2 cells (58) and hBD2 (human ß-defensin-2) in several epithelial cells (59). In the latter study induction with a mutated NOD2 failed to induce HBD2. This fits the concept that the NOD2 mutations in Crohn's disease share a signaling defect, the most pronounced occurs in the frameshift mutation 1007*fs*. Quite strikingly, NOD2 is also a directly active antibiotic and this action is also compromised by these mutations (60). However, the relevance of this mechanism *in vivo* is unclear.

When ileal α-defensins were related to the NOD2 status of the patients, in a first study (40) their expression was particularly low in those with mutations. In a second study these results were confirmed in a different cohort and the most pronounced effect was noted in the patients with the frame shift mutation (41). This was not observed in an Australian study (45) and also not in the pediatric study comparing the older and younger children (47). On the other hand, in the ileostomy patients (46) HD5 levels in the effluent of NOD2 homozygotes and compound heterozygotes were the lowest observed in the cohort. Looking at Paneth cell morphology, Crohn's patients carrying at least two NOD2 mutations exhibited an increased number of abnormal granules in Paneth cells (61). Finally, following small bowel transplantation, with 35% of the patients possessing NOD2 polymorphisms, rejection was characterized by decreased expression of Paneth cell antimicrobial peptides in the NOD2 mutant recipients, prior to the onset of inflammation (62). Finally, it has been repeatedly demonstrated that the NOD2 genotype impacts on the ileal microbiome in Crohn's disease (63). It seems likely but is unproven that this alteration is mediated by defensins. Unfortunately, in experimental animals the findings are similarly controversial (64, 65) and NOD2^−/−^ mouse organoids were not impaired in α-defensin expression (66). In contrast, in NOD2 deficient mice helicobacter hepaticus induced ileal granulomatous inflammation and this was reversed by transgenic expression of α-defensins in Paneth cells (67). Thus, NOD2 may well be important for Paneth cell defensin expression or secretion in mouse and man but the issue is not yet resolved.

Another risk gene identified in genome wide association studies is ATG16L1 (68) and this moved autophagy into the limelight. Autophagy is a process of degradation and recycling of cellular components, reducing cellular stress, but also of degrading bacterial components upon entry into the cell. It operates through the encapsulation of organelles and cytoplasm as well as bacteria within a membrane-bound organelle, termed the autophagosome (69). In a similar sequence of events to NOD2, next it was demonstrated that ATG16L1 and ATG5, another autophagy protein, play key roles in intestinal Paneth cells (70). ATG16L1 and ATG5 deficient or defective Paneth cells in both mouse and man exhibited striking abnormalities in the granule exocytosis pathway. During an infection lysozyme may be rerouted via secretory autophagy as an alternative secretory pathway and this is also affected in the ATG16L1 mutated mouse (71). At the same time some injury signals like acute phase reactants and adipocytokines were enhanced. When combined with a murine norovirus there was enhanced pathology following administration of toxic dextran sodium sulfate (72). Finally, the group succeeded in introducing the defective human Atg16L1 T300A variant gene into the mouse and again observed abnormalities in Paneth, but also in goblet cells (73). In human epithelial cells the variant impaired autophagy of *S. typhimurium* (74). Most importantly, however, it was demonstrated in Crohn's disease patients that genetic variants synthesize to produce Paneth cell phenotypes of Crohn's disease: i.e., the granule defects were more pronounced it the patient carried multiple NOD2 and ATG16L1 risk genes (61). Moreover, high proportions of abnormal Paneth cells were associated with shorter time to disease recurrence after surgery. The additive action of these genes is not surprising because NOD2 recruits ATG16L1 to the plasma membrane at the bacterial entry site and mutant NOD2 fails in this regard (75). Quite surprisingly, in Japanese patients there was a similar number of defective Paneth cells as in American patients, but this phenomenon was related to LRRK2 rather than ATG16L1 polymorphisms (76). LRRK2 is known to help sort lysozyme in cooperation with NOD2 and is also suppressor of autophagy: both processes may affect Paneth cell morphology (77).

Next, the focus turned from autophagy in Paneth cells to endosomal stress. This leads to accumulation of unfolded proteins within the endoplasmic reticulum (ER) lumen and a response directed by the receptor inositol-requiring enzyme 1 (IRE-1) which double-cleaves mRNA for XBP-1 (X-box binding protein 1) synthesis (78). This splicing activates XBP-1 to induce the unfolded protein response and, if it fails, cellular apoptosis is induced. For example, ischemia/reperfusion or obesity may lead to ER stress but there is also evidence that inflammatory bowel disease mucosa is “ER-stressed” (79). Moreover, the group reported that XBP-1 knockout mice exhibit loss of Paneth and goblet cells, reduced antibacterial activity and spontaneous enteritis. To complete the picture, and similar to NOD2 and ATG16L1, there was a clear-cut genetic link of hypomorphic XBP-1 polymorphisms to IBD. In elegant studies with single or double ATG and/or XBP-1 knockout mice it was demonstrated that *both* pathways affect and partly compensate each other. The combination of these genetic defects in the single mouse at last established the Paneth cell as a site of origin for intestinal inflammation (80). Further evidence that both pathways are interlinked is based on the observation that defective ATG16L1-mediated removal of IRE1α drives Crohn's disease like ileitis in the mouse (81). Finally, both pathways are involved in interleukin-22 signaling (82), a classical epithelial-protective cytokine. Novel findings now suggest that IL-22 actually orchestrates a pathological endoplasmic reticulum stress response and may also have deleterious facets (83).

Other genetic links of relevant Paneth cell genes to Crohn's disease are KCNN4 (84) and the Wnt factors TCF4 (85) and LRP6 (86). In addition, an unbiased genetic screen may well unravel further links as demonstrated recently (76).

## The Paneth Cell And Non-Genetic Links To Crohn'S Disease

Another important role in Paneth cell survival is played by caspase-8 which, if knocked out, induces TNFα-induced epithelial necroptosis and terminal ileitis. Its knock-out is also associated with lack of Paneth cells and reduced numbers of goblet cells (87). Accordingly, caspase-8 is essential to maintain intestinal barrier function and restrict pathogen colonization during *S. typhimurium* infection (88). Interferon lambda was recently shown to promote Paneth cell death in mice and is increased in inflamed ileal tissue in patients with Crohn's disease (89). Interestingly, glucocorticoids and tofacitinib, in current use in IBD, prevented Paneth cell death. Recently, it was described that also patients with inherited caspase-8 deficiency may develop intestinal inflammation but the role of caspase 8-genetics in Crohn's disease is not fully established (90).

An overarching factor affecting inflammatory response, amino acid metabolism, autophagy and also endoplasmic reticulum stress is ATF4 (activating transcription factor 4). Its levels were significantly decreased in inflamed mucosa of IBD patients and its deletion in mice was associated with diminished Paneth cell defensins (91). It should be emphasized, however, that although non-genetically deleted animal-models of terminal ileitis like the SAMP1/YitFc mouse also exhibit Paneth cell alterations (92), not all Paneth cell defects lead to spontaneous inflammation. In some models of ileitis the defective antibacterial system may be secondary to dysbiosis (93). In a very recent report, it was elucidated elegantly that even the Paneth cell specific knockout of prohibitin 1 triggers Paneth cell defects and ileitis in the mouse (94). Prohibitin 1 is not genetically linked to IBD but mitochondrial dysfunction and low levels of this mitochondrial protein have been observed. Interestingly, some species like the pig, not the cleanliest animal on earth, appear to perform quite well without Paneth cells.

## The Paneth Cell And Environmental Risk

In a recent review of metaanalyses several environmental risk factors for Crohn's disease were reevaluated and confirmed including smoking, antibiotic exposure, and vitamin D deficiency (95). All of these three factors impact on Paneth cell function and this link may represent a plausible mechanism of risk increase. For example, exposing mice to intragastric smoke condensate leads to alterations of ileal Paneth cell granules, antimicrobial peptide production and a reduction of bactericidal capacity (96). In Crohn's disease patients the combination of tobacco smoking and the ATG16L1 polymorphism combine to trigger Paneth cell defects and apoptosis (97).

Acute antibiotic treatment is known to decrease the protein level of lysozyme and of RegIIIγ as well as the mRNA level of α-defensin 5 (98). However, the long-term effects of “earlier in life” antibiotic treatment are unknown and therefore the analogy to patients with antibiotics in childhood and later Crohn's disease is speculative. Also, in animal models vitamin D deficiency together with high-fat feeding reduces α-defensin 5 and its activator MMP 7, similar to vitamin D receptor knockouts (99). Obese individuals exhibit decreased jejunal levels of HD5 and lysozyme, whereas Paneth cell numbers were unchanged (100). Thus, Paneth cell problems are not necessarily specific for Crohn's disease. Finally, chronic ethanol feeding also reduced α-defensin 5 in the mouse intestine (101) and possibly zinc deficiency plays a negative role in this context (102). Remarkably, in some of these circumstances (99, 101) oral administration of HD5 reversed the pathological changes. However, at least alcohol consumption is not an established risk factor for Crohn's disease, whereas zinc and vitamin D deficiency may well occur.

Finally, the microbiome may play a major role because bacteria (103), Listeria and Salmonella in particular (104, 105), as well as parasites like toxoplasma (106) and even viruses (107) all interact with Paneth cell physiology. It is common that patients report on an episode of gastrointestinal infection prior to developing IBD but this is not, to the best of our knowledge, an established link. Innate host defense, of course, is opposed to these infections but also “sculpts” the local commensal microbiome (30): as a consequence, Paneth cell defects may induce dysbiosis (108–110). However, it still remains an open question whether this dysbiosis is the hen or the egg, or both, with respect to the inflammatory process (6, 111). A detailed discussion of these host vs. microbiome issues is beyond the scope of this review but it is quite conspicuous that adherent-invasive *E. coli* associated with Crohn's disease are resistant to both α- and ß-defensins (112).

## Conclusion

**Figure 1:**
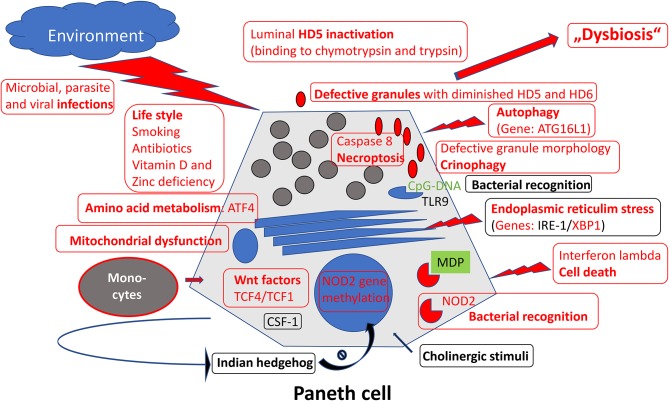
Overview of mechanisms regulating Paneth cell function and morphology. Those defective in ileal Crohn's disease were labeled in red.

After the first hints of a Paneth cell role in ileal Crohn's disease (19, 40), the Paneth cell as the key cell of defensin production in the small intestine proved to be an exciting focus of IBD-research, in recent years and in many respects. The various defects of this specialized cell in ileal Crohn's disease (Figure 1), in particular the (necessarily primary) genetic defects, have convinced many in the field that deficient defensins may represent one of the key events in triggering the disease (7, 113). The microbiome directed immune response and the stable localization over time is unlikely to be explained by a mere T-cell overresponse and, therefore, unlikely to represent an autoimmune disease (107). Future studies on the regulatory network of Paneth cells, maybe like those reported recently, using transcriptomics approaches may delineate additional complexity in these already remarkably versatile cells (114). Finally, and this is what counts for the patients: if the chance to substitute for mucosal defensins by systemic or oral administration, as mentioned above, really works out, this originally unlikely hypothesis may lead to a promising new therapy both in Crohn's disease (115, 116) as well as in intestinal graft vs. host disease (117).

## Author Contributions

Both authors have made substantial, direct and intellectual contribution to the work and approved it for publication.

### Conflict of Interest

JW holds different patents on defensin treatments in different diseases including inflammatory bowel disease, asthma, and metabolic syndrome. ES declares that he serves as a consultant to Curevac, Tübingen, Germany.
